# Indigestion in Infants and Children1Read before the Cattaraugus County Medical Society at Olean, N. Y., August 2, 1898.

**Published:** 1898-09

**Authors:** Clarence King

**Affiliations:** Machias, N. Y.


### BUFFALO MEDICAL JOURNAL.

VOL. XXXVIII. SEPTEMBER, 1898. NO. 2

### Original Communications.

### INDIGESTION IN INFANTS AND CHILDREN.[1]

BY CLARENCE KING, M. D., Machias, N. Y.

THE three score and ten years of human life may well be divided into three equal periods by which we have about twenty-three years devoted to the process of development and the acquisition of knowledge, twenty-three years to the performance of our life-work and twenty-three years to decay. It is generally during this second period or the earlier part of the third that those achievements have been accomplished by men in all ages which have sent their names down to posterity as moral, intellectual or physical giants. But the attainments of this period presuppose a healthy growth, bodily and mental, during the first period and it is, therefore, evident that the first few years of life are, after all, the most important of our existence. If they go wrong the chances are that our whole life will be warped and unproductive of the greatest results.

In the attainment of physical growth, with which we are at present concerned, digestion and respiration constitute our two post-natal methods. The part which respiration takes in feeding us with oxygen for the nutrition of the blood and hence for the regeneration of the tissues concerns us not at this time, and we dismiss it with this passing notice. Digestion is our theme, or rather its opposite, as applied to the period of most active growth in infancy and childhood.

Digestion may be tersely defined as that process by which food is converted into nutriment, and assimilation as the transformation of such nutriment into the tissues and fluids of the body. Indigestion, on the other hand, not only expresses the failure of this metamorphosis of food, but also precludes the possibility of assimilation hence malnutrition results.

1. Read before the Cattaraugus County Medical Society at Olean, N. Y., August 2, 1898.

Digestion in infants differs materially from digestion in adults, owing to the imperfect development of some of the organs concerned in the process, and in infants the liability to indigestion is consequently greater than in older persons. For comparison let us briefly consider normal digestion in adults and in infants, after which we can more intelligently study its derangements.

*Physiology of digestion in adults.*—Physiology teaches us that digestion begins in adults in the bucchal cavity by the conversion of starch into glucose through the action of the salivary secretion. But this function of the salivary glands is generally regarded as secondary to the mechanical one of moistening and thus perparing the bolus of food for easy deglutition. The esophagus has never been regarded as a digestive organ, but simply as a channel from one part of the digestive system to another; food passes through it too rapidly to be changed in the least during transit and it is devoid of any important secretion. The stomach, then, receives the food but little altered in its chemical characteristics. In man this organ occupies nearly a horizontal position in the upper abdomen and, according to the recent researches of Dr. Cannon, is essentially a double organ, with two separate and distinct functions. The greater or cardiac pouch of the stomach is the great reservoir which receives the food and stores it until the digestion of starch is completed by the action of the salivary secretion with which it has been intimately mixed. This part of the stomach is but sparingly supplied with circular muscular fibers and peristaltic action is consequently absent, or at least very feeble. But it contains peptic glands and mucous follicles and these secrete an acid fluid, which neutralises the alkaline saliva but not, under normal conditions, until the starch in the food has been converted into glucose. After this change has occurred the food is passed into the next portion of the stomach for its second mastication and mixture with digestive agents. Its presence here excites active peristaltic movements of this part of the stomach, which divide again and again the morsels and convert the whole into one homogeneous pulp. The particles of food in contact with the stomach wall are forced forward by the successive peristaltic waves until they reach the pylorus, which not being open, turns them back again through the center of the mass and this circuit is repeated over and over again until they are finally passed into the intestine. Albumin, fibrin and casein are readily dissolved and digested in the stomach, but fats and sugars require mixture with the intestinal fluids. Perhaps the most important of these is the pancreatic juice which emulsifies fats and has an important function in the digestion of albuminoids, gluten and casein, after they have been acted upon in the stomach. The bile and the secretion from the intestinal tubules have important functions, but their uses in digestion have not been as fully cleared up as the other fluids. Certain it is, they act on starch; and albumin and meat are more or less digested by them.

*Physiology of digestion in infants and children.*—With adults, solid food of a mixed nature normally forms our chief sustenance. But the salivary glands are poorly developed in infants and the mouth is consequently comparatively free from saliva, which makes it difficult for them to swallow solid food or digest starch. Hence, their food should be in a liquid form and comparatively free from starch. Again, the stomach occupies a different relative position than it does in the adult, being more nearly perpendicular. It is also normally smaller in size and the gastric glands are not as well developed. Fluids are not retained in the stomach, except for a very brief period, but are passed almost immediately into the bowel below. For these reasons the stomach should be regarded in infants as the dilated lower end of the esophagus with but comparatively little function in digestion; while in adults it is the dilated upper end of the intestine with an important function. But the unique operation, recently performed by Schlatter, of Zurich, proves beyond a doubt that the stomach is not a necessary organ even in the adult, but is rather one of convenience. True, the infant stomach has a function in the digestion of casein, but this has probably been over-estimated, while the importance of intestinal digestion is not fully appreciated. Human milk contains about 30 per cent. of casein, which is readily dissolved and digested by the infant stomach; but casein may also be digested in the intestine by the combined action of the intestinal fluids. Fat exists in human milk in about the same proportion as casein and in such quantities is readily digested by the pancreatic secretion, but this amount can not be much increased without its appearance in the stools. The liver in infants is relatively larger than in adults and is richer in blood. Its secretion is poorer in certain biliary acids, which in the adult tend to neutralize the action of pepsin soon after its passage with the food into the intestine. Hence, the process of digestion begun in the stomach by the action of pepsin may be continued in the intestine by this same ferment, in much the same way as the digestion of starch is accomplished in the stomach of adults by the salivary secretion. And, lastly, the intestinal channel is longer in infants in proportion to their size than in adults and its muscular activity is less, thus insuring the retention of its contents for a longer time within the influence of its digestive action and absorbent powers.

*Etiology.* —The first few months of life are devoted solely to bodily growth and it is not until about the sixth month that mental activity is aroused in the least. During this time errors of digestion are common, generally due to the mental incapacity of the nurse in the proper selection of the food or in the manner of giving it; but hot weather, dentition or accompanying disease may be predisposing factors. Bottle-fed babies are more liable to indigestion than those nourished at the breast; and those who are allowed solid or improper articles of food at too early a period are more endangered than either. In older children errors in hygiene, especially in the matter of dress, the pernicious habit of eating between meals, the indulgence in tea and coffee and sweet cake and the eating of candy and peanuts are frequent causes. As a result indigestion follows, diarrhea supervenes, malnutrition ensues and our infant mortality is swelled beyond reason.

Indigestion in infants and children may be, as in adults, either acute or chronic; when acute, the stomach is generally at fault, but when chronic it is usually due to intestinal causes. Every nurse is familiar with the vomiting or regurgitation of milk which follows too free nursing either at the breast or the bottle. If this occurs immediately the milk is ejected in a fluid form and it can hardly be called indigestion as it represents the effort of the first or cardiac portion of the stomach to empty itself into the part below, as it normally should do, soon after nursing. This vomiting, like the escaping steam from a safety valve, prevents accident, *i. e.*, indigestion, and is not of itself disease. But if vomiting is delayed for a few minutes and the ejecta contains much coagulated casein then we may assume either a state of hyperacidity of the stomach or some derangement in the solvent power of its secretion. Of course, this distinction is often arbitrary and cannot always be made clinically, as the two conditions frequently merge into or overlap each other.

*Symptoms of acute indigestion.*—The symptoms of acute indigestion in infants generally include vomiting of curdled milk or solid pieces of undigested food, fever, disturbed sleep and often diarrhea. If the exciting cause is in the food and this is vomited early and the stomach thoroughly emptied the attack may be cut short and the other symptoms not appear. Otherwise fever quickly follows, the cheeks become flushed, drowsiness supervenes, but with disturbed sleep and that condition, always popular with the laity and known as "worms"

is presented. Under little or no treatment this may pass away in a few hours with a copious or a very offensive passage from the bowels, or if aggravated by hot weather or other causes which enfeeble the powers of resistance, an acute dyspeptic diarrhea may result, which in its turn may be followed by enterocolitis. In severe cases, or in children of a neurotic temperament convulsions may usher in or accompany the attack. Older children often have headache, a coated tongue and tenderness or pain in the epigastrium, while the abdomen may be distended and tympanitic. These symptoms may all subside within a few hours or last a day or two, after which the child rapidly regains its normal health. But these attacks may be repeated again and again until the character of the food or the climatic or sanitary conditions are changed, or the stomach has become better developed and more tolerant to abuse, or death from inanition results.

*Treatment of acute indigestion.*—The treatment of acute indigestion consists in the first place of the thorough evacuation of the stomach and intestines. If free emesis has not already occurred ipecac should be given and this followed by calomel or castor oil or other cathartic, in palatable form. Ordinarially, vomiting has occurred before the physician is called and occasionally it persists and causes much alarm to the anxious mother. In such cases we should seek to allay gastric irritation by means of a sinapism or spice plaster, applied over the stomach and the administration, at frequent intervals, of small doses of ipecac, or a few drops of a mixture of equal parts of lime water and cinnamon water. The child should be kept perfectly quite in its crib or nurse's lap and all food and other medicines withheld from the stomach. But if restlessness is prominent an opiate may be given by the rectum, suspended in starch water and frequent sponging of the face and extremities, with tepid water, may be practised. In older children vomiting is not as common, but the offending food passes into the bowel and causes diarrhea. If seen early, these cases should promptly have an emetic, but if diarrhea has developed they must be treated by laxatives and stimulants and occasionally by digestants in accordance with the principals generally laid down for the treatment of dyspeptic diarrhea.

*Etiology of chronic indigestion.*—Chronic indigestion, I have stated, is usually caused by intestinal errors, but such is not always the case. Thus, dilatation of the stomach from atony of the gastric walls, which is a frequent complication of advanced rickets or adynamic disease may be an occasional cause. Habitual congestion of the mucous membrane, as is often induced in children of the poor by the use of beer or unsuitable food is a common cause in some localities ; and the scrofulous and gouty or lithemic diathesis may also produce the disease. Habitual over-feeding taxes to the utmost the powers of the whole digestive tract and the slight irritation from this cause induces increased mucous secretion from the intestinal membrane, which according to Dessau, is rich in mucin and forms a favorable culture medium for the bacteria normally present in the intestine. This causes putrefaction of the intestinal contents with liberation of gases and the possible formation of toxins, with a mild auto-intoxication, sufficient to cause retention of the digestive secretions or even peristaltic paralysis. And, finally, the frequent neglect of sanitary precautions, the fatigue following violent exercise and the depressing effects of the acute diseases during this period of rapid growth and development all predispose to irregularities in the nutritive process, which acting slowly but constantly, often cause chronic indigestion.

*Symptoms of chronic indigestion.*—Vomiting is not a common symptom in the chronic form of indigestion, except as it is produced by an acute attack following some extraordinary cause. But nutritive defects are apparent throughout the whole system ; the skin is pale, the muscles flabby, the circulation poor and the hands and feet are often cold and blue. In advanced cases there is loss of flesh and the eyes are sunken and discolored. The appetite may remain good, but more often it is fickle. The tongue is moist and often covered with a thin gray coating, but denuded of epithelium in sharpely defined patches of variable sizes and shapes. These children are languid and easily tired, at night they are restless and throw themselves in bed, kick off the bed covers and frequently have attacks of night terrors. Constipation is common, or constipation may alternate with diarrhea, the stools sometimes being mixed with mucus. Frequently, attacks of more or less severe abdominal pain occur at irregular intervals and the abdomen is distended with gas, and sometimes tender to pressure. The urine is high colored and loaded with phosphates and may contain uric acid in excess. Often it is passed during sleep, or at the moment of waking and may be the first symptom which induces the parents to consult a physician. Occasionally, attacks of fever occur, or syncope, or cough, or rapid breathing, or palpitation of the heart may divert attention from the real disease. But a microscopic inspection of the stools should help to clear the diagnosis, for it will invariably show shreds of undigested food and many oil globules.

In young infants the symptoms are essentially the same as in older children. They nurse eagerly when given the bottle or are put to the breast, but do not sleep quietly afterward; they jump and cry out and have to be rocked. Vomiting sometimes happens, as in acute indigestion, but is not frequent. They are fretful and want to be held and do not thrive well. They rub the nose and put the fingers in the mouth and if fever is present this gives rise to the belief that they are cutting teeth or that intestinal parasites are present. Usually they are constipated and the stools are passed in hard balls, or they may be pasty in consistence and light yellow in color. Sometimes prolapse of the rectum occurs from severe straining or the use of drastic purgatives. They have violent crying spells and attacks of colic are frequent and flatulency and eructation of gas generally occur. These symptoms may all persist for a long time until treatment or a radical change in the feeding or surroundings of the child gradually brings about a change for the better or the asthenia ends in death.

*Treatment of chronic indigestion.*—The treatment of chronic indigestion must begin with radical changes in the matter of food and feeding. Infants at the breast should be nursed regularly at intervals, varying during the day from an hour and a half to three hours, according to age, but at night not more than half as often. If the nurse becomes pregnant again or menstruation has returned, or there is other good reason to suspect the breast milk as the cause of the indigestion it may be well to substitute cow's milk properly diluted. I am not much of an advocate of artificial baby foods, yet doubtless there are cases where they have saved life after milk has proved a failure. Next, the bowels should be attended to and constipation removed. This sometimes is one of the most perplexing questions connected with the treatment of the disease and almost every laxative has had its advocates. Calomel in doses from 1-20 to 1-10 grain, four to six times a day, serves both as a laxative and as an antiseptic, but it should be discontinued for a few days, at least every fortnight. Hydriatics, as recommended by Baruch, and massage of the abdomen may be of service in some cases, and occasionally hot water injections may be given, but their habitual use tends to still further paralyze the bowel and make matters worse. The personal hygiene and sanitary surroundings should be attended to, older children should be encouraged to take outdoor exercise and infants should be wheeled by the nurse, or better, removed to the mountains or sea shore during hot weather. Cool sponging should be practised daily and special care exercised to prevent chilling of the body, especially during digestion. As aids to digestion we may employ diastase, pancreatin or pepsin, my preference being for the first two, but frequently I give pepsin and pancreatin combined. Theoretically, this combination is unscientific, but clinically it is frequently more satisfactory than either drug separately. Hydrochloric acid is sometimes useful in older children, but should seldom be given infants. Some authors advise the use of predigested foods, but as their use supplants normal digestion they should not be used except for brief periods to bridge over an emergency I prefer the digestion shall take place in the body, even if it has to be stimulated by digestants. Nux vomica or strychnia should also be employed in appropriate doses and the artificial digestants gradually reduced and withdrawn as early as possible.

*Special indications for treatment.*—The special symptoms or accompanying disorders often require active treatment. Of these the most frequent and distressing are flatulency and colic. It is not well to give opiates in this disease, but occasionally it seems unavoidable. High rectal injections of hot water will often cause the expulsion of gas with relief of pain and hot applications to the abdomen, or cold wet compresses, may accomplish the same result. Bismuth, either the subnitrate or subgallate, is one of the best intestinal antiseptics we have and should be given whenever flatulency is present or skin eruptions occur. Anemia, of course, calls for iron or cod liver oil, but these are difficult to digest and should not be given unless required. Restlessness and night terrors demand bromides, and intestinal parasites, when actually present, call for anthelmintics. Of these Dessau prefers spigelia, combined with senna, but santonin seems to be the one most universally employed. Other complications may be present and should be treated according to the dictates of reason and the accepted therapeutics of our art.

